# Identification of Potential Pathogenic Super-Enhancers-Driven Genes in Pulmonary Fibrosis

**DOI:** 10.3389/fgene.2021.644143

**Published:** 2021-05-12

**Authors:** Hang Li, Caiping Zhao, Zeli Li, Kainan Yao, Jingjing Zhang, Wenwen Si, Xiaohong Liu, Yong Jiang, Meiling Zhu

**Affiliations:** ^1^Central Lab, Shenzhen Bao’an Traditional Chinese Medicine Hospital (Group), Guangzhou University of Chinese Medicine, Shenzhen, China; ^2^Department of Respiratory, Shenzhen Bao’an Traditional Chinese Medicine Hospital (Group), Guangzhou University of Chinese Medicine, Shenzhen, China; ^3^Department of Pathology, Shenzhen Bao’an Traditional Chinese Medicine Hospital (Group), Guangzhou University of Chinese Medicine, Shenzhen, China; ^4^Department of Respiratory, The First Affiliated Hospital of Guangzhou University of Chinese Medicine, Guangzhou, China; ^5^Department of Respiratory, Shenzhen Hospital of Integrated Traditional Chinese and Western Medicine, Shenzhen, China; ^6^Traditional Chinese Medicine Innovation Research Center, Shenzhen Hospital of Integrated Traditional Chinese and Western Medicine, Shenzhen, China

**Keywords:** pulmonary fibrosis, super-enhancer, fibroblast, transcription factor, LTBP2

## Abstract

Abnormal fibroblast differentiation into myofibroblast is a crucial pathological mechanism of pulmonary fibrosis (PF). Super-enhancers, a newly discovered cluster of regulatory elements, are regarded as the regulators of cell identity. We speculate that abnormal activation of super-enhancers must be involved in the pathological process of PF. This study aims to identify potential pathogenic super-enhancer-driven genes in PF. Differentially expressed genes (DEGs) in PF mouse lungs were identified from a GEO dataset (GDS1492). We collected super-enhancers and their associated genes in human lung fibroblasts and mouse embryonic fibroblasts from SEA version 3.0, a network database that provides comprehensive information on super-enhancers. We crosslinked upregulated DEGs and super-enhancer-associated genes in fibroblasts to predict potential super-enhancer-driven pathogenic genes in PF. A total of 25 genes formed an overlap, and the protein-protein interaction network of these genes was constructed by the STRING database. An interaction network of transcription factors (TFs), super-enhancers, and associated genes was constructed using the Cytoscape software. Gene enrichment analyses, including KEGG pathway and GO analysis, were performed for these genes. Latent transforming growth factor beta (TGF-β) binding protein 2 (LTBP2), one of the predicted super-enhancer-driven pathogenic genes, was used to verify the predicted network’s accuracy. LTBP2 was upregulated in the lungs of the bleomycin-induced PF mouse model and TGF-β1-stimulated mouse and human fibroblasts. Myc is one of the TFs binding to the LTBP2 super-enhancer. Knockout of super-enhancer sequences with a CRISPR/Cas9 plasmid or inhibition of Myc all decreased TGF-β1-induced LTBP2 expression in NIH/3 T3 cells. Identifying and interfering super-enhancers might be a new way to explore possible therapeutic methods for PF.

## Introduction

Pulmonary fibrosis (PF) is typically a chronic, prolonged disease process. It can originate from known injuries (such as radiation therapy or toxic substances), be secondary to other diseases (such as connective tissue diseases), or happen without a specific reason (idiopathic pulmonary fibrosis, IPF; Haston et al., 2005). This disease is characterized by excessive proliferation and activation of (myo) fibroblasts, exaggerated extracellular matrix (ECM) deposition, and destruction of lung structure (Wolters et al., 2014). Even though anti-fibrotic drugs (such as nintedanib and pirfenidone) have been used in the clinic for this disease, the mortality is still high (King et al., 2014; Richeldi et al., 2014). There is an incomplete understanding of underlying pathological mechanisms (Lawrence and Nho, 2018). Many cells are involved in the pathological process of PF. Among them (myo) fibroblast plays an important role (Rosenbloom et al., 2017). Thus, abnormally activated (myo) fibroblast is usually the therapeutic target for researching drugs.

An enhancer is a short region of DNA bound by proteins (activators) to activate gene transcription. It can actively regulate gene expression in time and space through either cis‐ or trans-interaction (Shlyueva et al., 2014). Recently, a new cluster of regulatory elements, called super-enhancer, is attracting scientists’ special interests. They are large clusters, 8–20 kb in length, containing active transcriptional enhancers, and rich in high-density key transcription factors (TFs), co-factors, and enhancers (Whyte et al., 2013). Super-enhancers are believed to play a critical role in promoting the expression of cell recognition genes and can be used to explain cell-type-specific expression patterns (Hnisz et al., 2013). In tumorigenesis, Alzheimer’s disease, diabetes, and many autoimmune diseases, pathogenic gene expressions are found to be highly correlated with the abnormal activation of super-enhancers (Lovén et al., 2013).

Myofibroblasts are thought to originate from either resident fibroblasts or epithelial-to-mesenchymal transition (EMT; Li et al., 2020). Since super-enhancers play a critical role in determining cell identity, growth, and transformation, we believe that super-enhancers must take part in the abnormal proliferation and activation of lung myofibroblast during the process of PF. However, the role of pathogenic super-enhancers in the pathological process of PF has not yet been reported.

This study aims to predict potential pathogenic super-enhancers in PF. Figuring out the role of super-enhancers in PF development may help us further explore this refractory disease’s pathogenesis and find new therapeutic targets for it.

## Materials and Methods

### Identification of Differentially Expressed Genes in Lungs of PF Mice

GEO^1^ is a public database that stores curated gene expression datasets. A GEO dataset (GDS1492) was used to obtain DEGs in the bleomycin-induced mice model of PF. Normal C57BL/6 J mice, including female and male, were set as the control group. C57BL/6 J mice that received bleomycin treatment were designated as a model group. The GEO2R tool was used to identify DEGs between two groups of samples. The *p* values were adjusted by the Benjamini-Hochberg method. DEG was defined as reading number control group < model group and a *p*-value < 0.05.

### Prediction of Potential Super-Enhancer-Driven Pathogenic Genes in PF

SEA version 3.0 is a network database that provides a comprehensive extension and update of the super-enhancer archive (Chen et al., 2020). Through collecting and analyzing public ChIP-seq data, the SEA website has identified super-enhancers and their associated genes in different cells and tissues from various species. We searched identified super-enhancers in human lung fibroblasts and mouse embryonic fibroblasts detected by H3k27ac ChIP-seq in SEA. The information of super-enhancers, including super-enhancer ID in SEA, genomic loci, length, associated genes, and TFs, was collected. A Venn diagram was generated to show the overlap between upregulated DEGs and super-enhancer-associated genes in human lung fibroblasts and mouse embryonic fibroblasts. The overlapping genes demonstrated the potential super-enhancer-driven pathogenic genes in PF. The protein-protein interaction (PPI) network of super-enhancer-driven pathogenic genes was generated by the STRING database.^2^ The relationship between TFs, super-enhancers, and associated genes in mouse embryonic fibroblasts was presented by a network using Cytoscape 3.7.1.^3^

### KEGG Pathway and GO Analysis

Overlapping genes were uploaded to Enrichr^4^ to perform the KEGG pathway and GO analysis. The KEGG pathway data, including term, count, percentage, and *p* value, were uploaded to the HiPlot website^5^ to form a bubble diagram. GO analysis included a cellular component (CC), molecular function (MF), and biological process (BP).

### Animal Experimental Protocol

The experiments were approved by the Care and Use of Experimental Animals Committee of Guangzhou University of Chinese Medicine and performed according to the National Institute of Health Guide for the Care and Use of Laboratory Animals. C57BL/6 male mice (ages 6–8 weeks; bodyweight 18–20 g) were purchased from the Beijing Hua Fu Kang Biotechnology Co. Ltd. and housed in clean facilities without specific pathogens. According to the previous report, a bleomycin-induced mouse model of PF was established (Shlyueva et al., 2014). Mice were divided into the control group and model group (*n* = 6 per group). On day 1, mice were anesthetized firstly, and 2.5 mg/kg of bleomycin (Macklin, Shanghai, China) or saline was instilled through the airways into the lungs. On day 21, after bleomycin instillation, the lung tissues were harvested for further examinations.

### Hematoxylin and Eosin and Masson’s Trichrome Staining

The lungs were fixed in 4% paraformaldehyde, embedded in paraffin, and then sliced (4–5 μm) for H&E and Masson’s trichrome staining. For H&E staining, sections were stained in hematoxylin for 5 min and then stained for 2 min at room temperature in eosin. Collagen in lung tissues was demonstrated by Masson’s trichrome staining kit (Solarbio, Beijing, China) according to the manufacturer’s instructions. The collagen fibers were stained blue. The muscle fibers, cytoplasms, celluloses, keratins, and red cells were stained red. The nucleus was blue-brown. H&E and Masson’s staining were photographed using a light microscope.

### Western Blotting

The lung tissues were lysed with a RIPA lysate (Sigma-Aldrich, St. Louis, MO, United States). The supernatant was centrifuged to extract the total protein. The protein concentration was determined using a BCA protein assay kit (Pierce, Rockford, United States). The protein was denatured by high-temperature treatment for 5 min after the addition of the loading buffer. Protein was separated on SDS-PAGE gels and transferred to PVDF membranes. The membranes were blocked with 5% BSA for 1 h at room temperature. After incubation with the *COL1A1* (CST, Danvers, MA, United States), *LTBP2* (Affinity Biosciences, Jiangsu, China), or β-tubulin antibody (CST, Danvers, MA, United States) overnight at 4°C, membranes were incubated with the secondary antibody for 1 h at room temperature. Immunoreactive bands were detected using a chemiluminescent substrate system and analyzed using ImageJ (NIH, United States).

### Hydroxyproline Content Measurement

The hydroxyproline content in mice’s lung tissues was analyzed using a hydroxyproline assay kit (Sigma-Aldrich, St. Louis, MO, United States). Approximately 10 mg of lung tissues from each mouse was used for detection. The operation process is done according to the manufacturer’s instructions and as previously reported (Liu et al., 2013).

### Real-Time Polymerase Chain Reaction

Total RNA of lung tissues or cells was extracted using an RNA simple Total RNA Kit (TIANGEN, Beijing, China). RNA was reverse transcribed into cDNA using TransScript® All-in-One First-Strand cDNA Synthesis SuperMix for qPCR (One-Step gDNA Removal; TransGen, Beijing, China). Real-time PCR was performed using TB Green™ Premix Ex Taq™ II Kit (Takara, Tokyo, Japan) on an LightCycler 480 real-time PCR system. All data were quantified using the 2-ΔΔCT method in relative quantification and normalized to GAPDH mRNA expression. The primer sequences of the target genes are listed in Table 1.

**Table 1:** Primers.

Gene	Forward primer (5'–3')	Reverse primer (5'–3')
mouse α-SMA	TCAGGGAGTAATGGTTGGAATG	GGTGATGATGCCGTGTTCTA
mouse COL1A1	AGACCTGTGTGTTCCCTACT	GAATCCATCGGTCATGCTCTC
mouse fibronectin	TCCTGTCTACCTCACAGACTAC	GTCTACTCCACCGAACAACAA
mouse GAPDH	AACAGCAACTCCCACTCTTC	CCTGTTGCTGTAGCCGTATT
mouse LTBP2	CTCGCTGAGTGGTAAGAAATACA	CTTGGCTCCTCCTTTCGTATC
human α-SMA	GATGGTGGGAATGGGACAAA	GCCATGTTCTATCGGGTACTTC
human COL1A1	CCTGTCTGCTTCCTGTAAACTC	GTTCAGTTTGGGTTGCTTGTC
human fibronectin	CTGAGACCACCATCACCATTAG	GATGGTTCTCTGGATTGGAGTC
human GAPDH	GGTGTGAACCATGAGAAGTATGA	GAGTCCTTCCACGATACCAAAG
human LTBP2	AAGGGAGGGTTGCATAACAG	GTTCTCCGATGGTGAGGTATAAG
mouse LTBP2 SE	TCCTTGGAGGGACAGCTCGGC	AGTTGCCCTTCCCCATAGGCAAC

### Immunofluorescence Staining

Lung tissues were fixed overnight with 4% paraformaldehyde and then sliced (4–5 μm) for immunofluorescence staining under a frozen state. The sections were blocked with 5% goat serum and 0.3% Triton X-100 in phosphate-buffered saline-Tween 20 (PBST) for 1 h. Then, the sections were incubated with collagen I (GeneTex, Irvine, CA, United States) and *LTBP2* (Bioss, Beijing, China) primary antibody at 4°C overnight and then washed and incubated with fluorochrome-conjugated secondary antibody for 1 h at room temperature. After washing the sections again, slides were mounted with the ProLong Gold Antifade reagent with DAPI, and images were captured using Cytation 5 (BioTek, United States).

### Cell Culture

Mouse embryonic fibroblasts (NIH/3 T3) and human fetal lung fibroblasts (HFL1) were purchased from the National Collection of Authenticated Cell Cultures (Shanghai, China). All cell lines were cultured in Dulbecco’s modified Eagle’s medium (DMEM; Gibco, Grand Island, NY, United States) containing 10% bovine calf serum (Tianhang, Zhejiang, China), 100 U/ml penicillin, and 100 μg/ml streptomycin (Gibco, Grand Island, NY, United States). Cells were maintained in a humidified atmosphere containing 5% CO_2_ at 37°C.

For real-time PCR analysis, the cells were seeded in six-well plates. After 80% confluency, NIH/3 T3 and HFL1 cells were treated with 5 ng/ml recombinant mouse or human transforming growth factor beta 1 (TGF-β1; R&D Systems, Minneapolis, MN, United States), with or without the myc inhibitor 10058-F4 (Selleck Chemicals, Houston, TX, United States) for 12 h. Then, total RNA was extracted.

### Genome Editing

Sequences of latent TGF-β binding protein 2 (*LTBP2*)-associated super-enhancer (*LTBP2* SE) in mouse embryonic fibroblasts were obtained from the SEA database. The position of *LTBP2* SE in the chromosome is mm10 chr12: 84783211–84876491. We screened sequences of LTBP2 SE, which has a motif that could be bound by myc TF, and designed a CRISPR/Cas9 plasmid that specifically targeted the site at mm10 chr12: 84876609–84876861. The CRISPR/Cas9 plasmid pSpCas9(BB)-2A-Puro(PX459) was manufactured by Hanbio Biotechnology (Shanghai, China). NIH/3 T3 cells were transfected with a CRISPR/Cas9 plasmid using the Lipofectamine 3000 reagent (Invitrogen, Carlsbad, CA, United States) and screened by puromycin-containing media according to standard transfection protocols.

### Genomic DNA Isolation and PCR

To assess the genome editing’s efficacy, the genomic DNA of NIH/3 T3 cells was extracted using the TaKaRa MiniBEST Universal Genomic DNA Extraction Kit version 5.0 (Takara, Tokyo, Japan). PCR was performed using Premix Taq™ (Ex Taq™ version 2.0 plus dye; Takara, Tokyo, Japan) according to manufacturer’s instructions. The primer sequences of the target gene (mouse *LTBP2* SE) were listed in Table 1. Then products were electrophoresed in a 1% agarose gel, and images were captured under ultraviolet light.

### Statistical Analysis

All data analyses were performed using the GraphPad Prism software (version 8.2.1, GraphPad Software, Inc.). Data are presented as mean ± standard deviation. The normality of values was tested with the Shapiro-Wilk normality test. Comparisons were made using one-way analysis of variance followed by Tukey’s test for multiple comparisons or using the nonparametric test (Dunn’s test), depending on the data distribution. Two-group comparisons were performed using a *t*-test. A *p*-value < 0.05 was considered significant.

## Results

### Prediction of Potential Super-Enhancer-Driven Pathogenic Genes in PF

**Figure 1:**
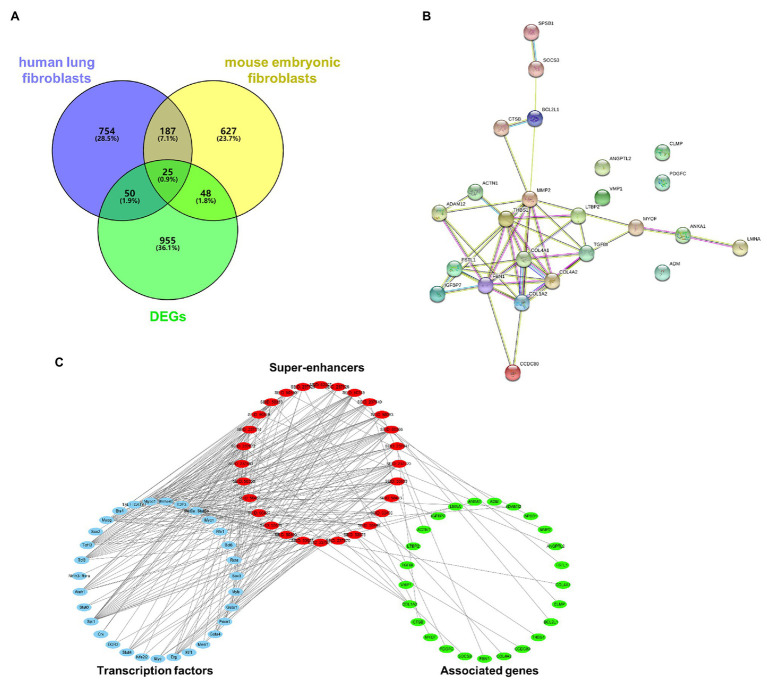
Prediction of potential super-enhancer-driven pathogenic genes in PF. **(A)** Venn diagram of upregulated DEGs in lungs of PF mice from a GEO dataset (GDS1492) and super-enhancer-associated genes in human lung fibroblasts and mouse embryonic fibroblasts from the SEA database. **(B)** A PPI network of the 25 super-enhancer-driven pathogenic genes was generated using the STRING database. **(C)** The interaction network of TFs, super-enhancers, and associated genes in PF.

A GEO dataset (GDS1492) was used to identify DEGs in the bleomycin-induced mice PF model. There were 1,085 DEGs upregulated in the PF model. There were 1,131 super-enhancer-associated genes identified in human lung fibroblasts in the SEA web and 1,035 super-enhancer-associated genes in mouse embryonic fibroblasts. Since activated super-enhancers drive high expression of targeted genes, we crosslinked these super-enhancer-associated genes with upregulated DEGs to predict PFs’ potential super-enhancer-driven pathogenic genes. A Venn diagram showed that there were 25 genes that overlapped (Figure 1A). These genes were considered as potential super-enhancer-driven pathogenic genes of PF. The PPI network between these super-enhancer-driven pathogenic genes was analyzed using the STRING database (Figure 1B). An interaction network of TFs, super-enhancers, and associated genes in PF was constructed (Figure 1C).

### KEGG Pathway and GO Analysis

**Figure 2:**
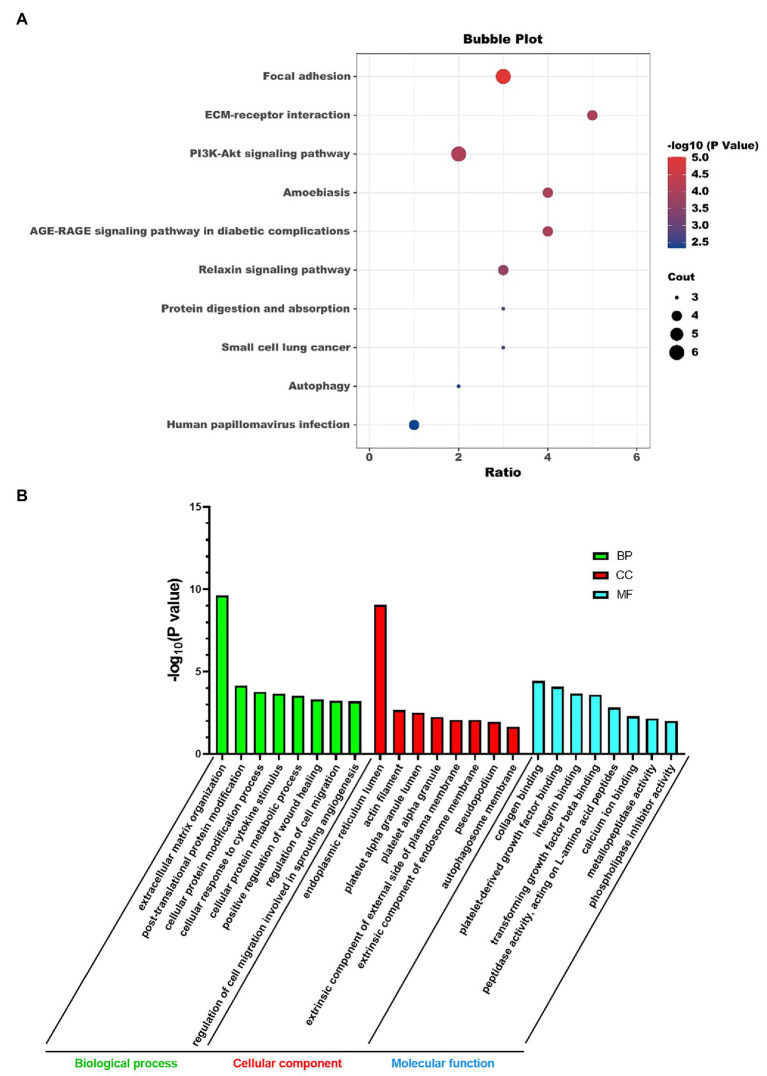
KEGG pathway analysis **(A)** and GO analysis **(B)** of super-enhancer-driven pathogenic genes in PF.

We did KEGG pathway and GO analysis for these super-enhancer-driven pathogenic genes. The primarily enriched pathways were focal adhesion, ECM-receptor interaction, PI3K-Akt signaling pathway, and so on (Figure 2A). GO analysis results, including BP, CC, and MF, are shown in Figure 2B.

### LTBP2 Was Highly Expressed in Lungs of Mice With PF

**Figure 3:**
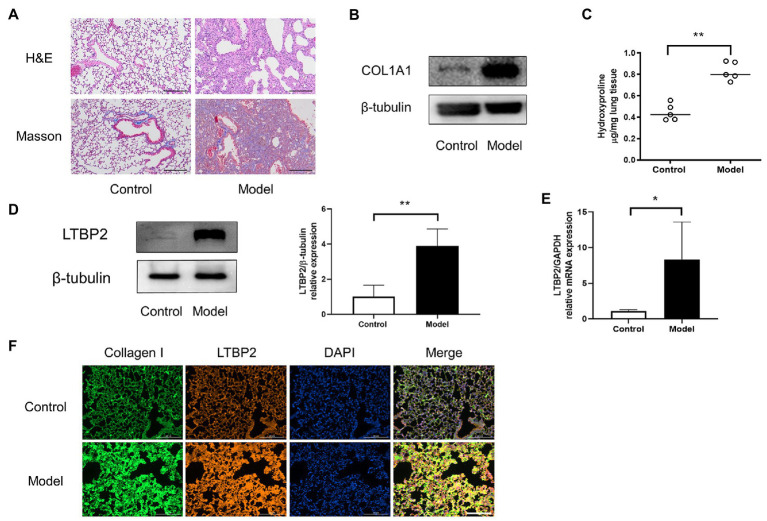
LTBP2 was highly expressed in lungs of mice with PF. **(A)** Representative images show H&E and Masson’s trichrome staining of lung sections from the indicated groups of mice. Scale bars: 100 μm. **(B)** Representative bands of western blots showing expression of COL1A1 and β-tubulin in lungs of indicated mice. **(C)** Quantitative analysis of hydroxyproline in lung homogenates from indicated mice. **(D)** Representative bands of western blots showing expression of LTBP2 and β-tubulin in lungs of indicated mice. **(E)** The mRNA expressions of a-SMA, COL1A1, fibronectin, and LTBP2 in lungs were detected by real-time PCR. **(F)** Co-immunofluorescence staining for collagen I (green), LTBP2 (red), and DAPI (blue) in bleomycin-treated mice. Original magnification, scale bars: 100 μm. Data presented as mean ± SD, *n* = 4–5. ^*^p < 0.05; ^**^p < 0.01.

Now we have predicted 25 potential super-enhancer-driven pathogenic genes of PF. Among them, we chose *LTBP2* to confirm our hypothesis. A PF mouse model was induced by bleomycin. H&E and Masson staining showed that the lung tissues’ structural integrity in the model group was destroyed (Figure 3A). At the same time, there was plenty of inflammatory cell infiltration and extensive deposition of fibrillary collagen. *COL1A1* protein is an important marker of organ fibrosis. *COL1A1* protein expression significantly increased in the lungs of the model group (Figure 3B). Hydroxyproline content is another essential indicator used to evaluate collagen metabolism and the fibrotic degree of an organ. After the bleomycin challenge, hydroxyproline content in the lungs significantly increased (Figure 3C). Subsequently, we confirmed that *LTBP2* protein and mRNA were highly expressed in PF mice’s lungs (Figures 3D,E). Double immunofluorescence staining also showed that collagen I and *LTBP2* staining were broadly positive in the fibrotic interstitium in PF lungs, and they had co-localization (Figure 3F).

### LTBP2 Was Highly Expressed in TGF-β1-Induced Myofibroblasts

**Figure 4:**
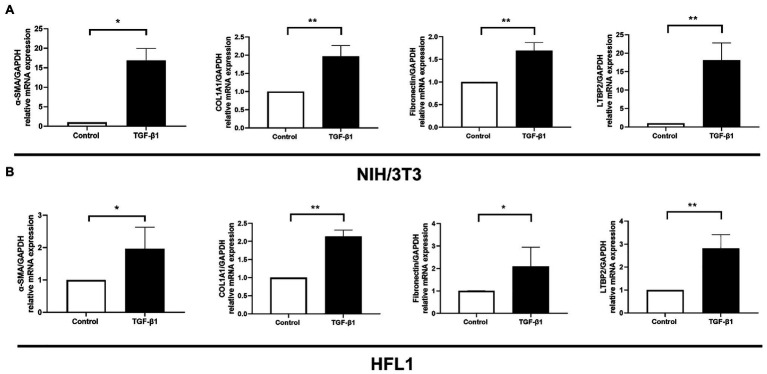
LTBP2 was highly expressed in TGF-β1-induced myofibroblasts. The mRNA expressions of a-SMA, COL1A1, fibronectin, and LTBP2 in mouse embryonic fibroblasts (NIH/3 T3; **A**) and human fetal lung fibroblasts (HFL1; **B**) were detected by real-time PCR after 5 ng/ml TGF-β1 stimulation for 12 h. Data presented as mean ± SD, *n* = 3–4. ^*^p < 0.05; ^**^p < 0.01.

It is well known that TGF-β1 cytokine is closely related to organ fibrosis (Kim et al., 2018). It is the master regulator of myofibroblast activation and ECM accumulation (Bartram and Speer, 2004). After TGF-β1 stimulation, fibrotic markers, including α-SMA, *COL1A1*, and fibronectin, all increased either in NIH/3 T3 or in the HFL1 cell line (Figures 4A,B). At the same time, *LTBP2* mRNA expression increased.

### Knockout of Super-Enhancer Sequences and Inhibition of Myc Decreased LTBP2 Expression

**Figure 5:**
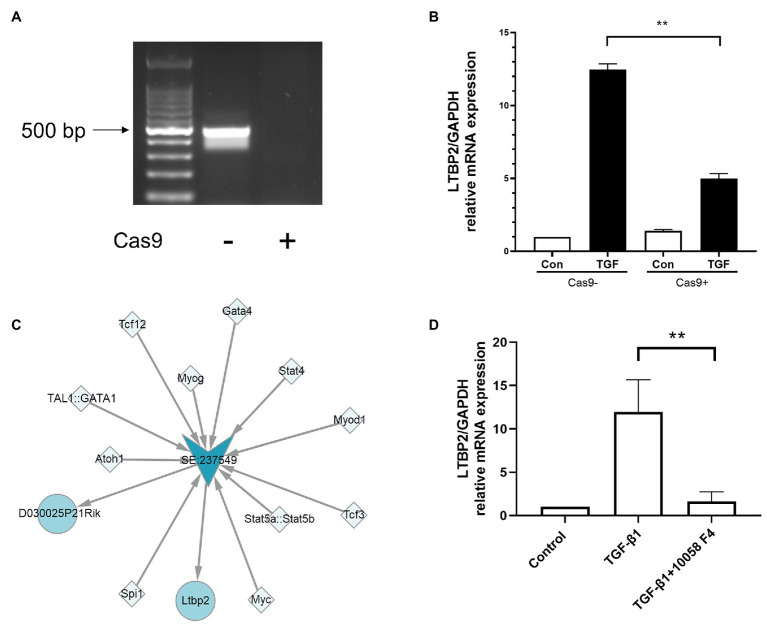
Knockout of super-enhancer sequences and inhibition of myc decreased LTBP2 expression. **(A)** Genomic DNA of NIH/3 T3 cells transfected with or without a CRISPR/Cas9 knockout plasmid was extracted. PCR was used to detect the expression of targeted genes. Products were electrophoresed in a 1% agarose gel, and images were captured under ultraviolet light. **(B)** The mRNA expressions of LTBP2 in NIH/3 T3 cells transfected with or without a CRISPR/Cas9 knockout plasmid were detected by real-time PCR after TGF-β1 stimulation for 12 h. **(C)** TFs binding the LTBP2 super-enhancer (SE:237549). Squares represent TFs. Arrows represent the super-enhancer. Circles represent targeted genes. **(D)** The mRNA expressions of LTBP2 in NIH/3 T3 cells were detected by real-time PCR after 5 ng/ml TGF-β1 and 20 μM myc inhibitor (10058-F4) treatment for 12 h. Data presented as mean ± SD, *n* = 3. ^**^p < 0.01.

To verify whether its super-enhancer regulates LTBP2 expression, we designed a CRISPR/Cas9 plasmid to knock out part of LTBP2’s super-enhancer sequences. It was shown that CRISPR/Cas9 plasmid successfully knocked out targeted sequences in NIH/3 T3 cells (Figure 5A). Surprisingly, super-enhancer knockout significantly inhibited TGF-β1-induced *LTBP2* mRNA expression (Figure 5B). Super-enhancers are driven by a cluster of TFs and co-factors. From the SEA database, we know that in the *LTBP2* super-enhancer of mouse embryonic fibroblasts, there are 11 TF binding domains (Figure 5C). Myc is one of the predicted TFs taking part in driving the *LTBP2* super-enhancer. It was proved that the inhibition of myc decreased *LTBP2* expression (Figure 5D), indicating that interfering pathogenic super-enhancers in PF may be a potential therapeutic method for this disease.

## Discussion

Myofibroblasts are the critical effector cells in PF (Li et al., 2020). They are responsible for the synthesis and deposition of ECM. Fibroblast differentiation into myofibroblast is one kind of change of cell identity. Activated pathogenic super-enhancers drive pathogenic gene expression, which is the potential pathogenesis of diseases (Wang et al., 2019). At present, only limited articles discuss the role of super-enhancers in PF. One study reported that T-box TFs, especially *TBX4*, are associated with super-enhancer-driven transcriptional programs underlying features specific to lung fibroblasts (Horie et al., 2018). Another one reported that *FOXL1*, a TF, has high transcripts of DNA hypomethylation and super-enhancer formation in lung fibroblasts (Miyashita et al., 2020). Due to super-enhancers’ close relation to cell identity‐ and fate-determined processes (Peng and Zhang, 2018), we speculate that super-enhancers must take part in the process of fibroblast activation in PF. Activation of related super-enhancers is the potential mechanism of high expression of DEGs in PF. Here, we predict the potential super-enhancer-driven pathogenetic genes in PF by overlapping with upregulated DEGs in PF mice and super-enhancer-targeted genes in mouse and human fibroblasts.

Several super-enhancer-driven genes, including *COL1A2*, *COL4A1*, *COL4A2*, and *FBN1*, belong to the ECM component. It is well known that collagen-rich ECM produced by lung fibroblasts will distort lung structure and seriously disturb the healthy gas exchange (King et al., 2001). The function of super-enhancers is reflected by their associated genes. Gene enrichment analysis helps us figure out what role those super-enhances-driven genes may play in the process of PF. Comprehensive analysis of the KEGG pathway and GO analysis results hints that super-enhancer-driven genes are mainly involved in regulating focal adhesion, ECM, and integrin.

Now, a regulatory network of TFs, super-enhancers, and associated genes in PF has been constructed. We chose *LTBP2* to verify the accuracy of our predicted network. *LTBP2* is an extracellular secretion protein that belongs to the fibrin/LTBP superfamily protein. It is mainly expressed in the lung, skin, and large blood vessels (Wang et al., 2018). *LTBP2* has various biological functions involving the ECM composition and plays a vital role in elastic fibers and cell adhesion (Enomoto et al., 2018). Interestingly, we detected high mRNA and protein expression of *LTBP2* in lungs of the PF mouse model and TGF-β1-stimulated mouse and human fibroblasts. A previous study has reported that *LTBP2* is secreted from lung myofibroblasts and may reflect the level of differentiation of lung fibroblasts into myofibroblasts in IPF (Enomoto et al., 2018). This hints that *LTBP2* is a biomarker of cell identity in myofibroblasts, consistent with the super-enhancer’s function.

However, whether the highly expressed genes are driven by activation of associated super-enhancers is still not clear. It needs to be further confirmed by experiments like CRISPR/Cas9 genome editing (Yoo et al., 2019). Thus, we designed a CRISPR/Cas9 plasmid to knock out part of *LTBP2* super-enhancer sequences. Surprisingly, super-enhancer sequences knock out inhibited TGF-β1-induced *LTBP2* mRNA expression in NIH/3 T3 cells. This further verified that *LTBP2* is transcriptionally regulated by associated super-enhancers.

Super-enhancers can recruit large numbers of transcriptional complexes, including activated TFs (Joo et al., 2019). According to data from the SEA website, myc is one of the TFs binding to the *LTBP2* super-enhancer. Studies have shown that c-myc is involved in the occurrence of multiple organ fibrosis, including renal fibrosis (Shen et al., 2017), PF (Yin et al., 2019), myocardial fibrosis (Zhang and Sun, 2019), lens fibrosis (Zhang et al., 2019), and liver fibrosis (Sharawy et al., 2018). We found that the myc inhibitor 10058-F4 also inhibited TGF-β1-induced *LTBP2* mRNA expression in NIH/3 T3 cells, further confirming the network’s accuracy of TF-super-enhancer genes.

When key TF is knocked down, super-enhancer-associated genes’ expression decreases faster than typical enhancer-associated genes (Lovén et al., 2013). This indicates that a super-enhancer has higher transcription activation and a higher interference sensitivity than a typical enhancer, making it a potential therapeutic target for diseases. Analysis of binding motifs of TFs confirms that the super-enhancer site is rich in cell-specific key binding motifs of TFs (Hnisz et al., 2013). Super-enhancers consist of clusters of enhancers densely occupied by key TFs and the mediator coactivator (Whyte et al., 2013). We have constructed a potential TF-super-enhancer-associated genes regulatory network of PF. Interfering key super-enhancers and key TFs may be the next step to explore the possible therapeutic methods for PF. Promisingly, some attempts have been made to use super-enhancers as a tool for disease treatment, such as small-molecule inhibitors and gene therapeutic approaches (Shin, 2018). For example, JQ1, a *BET* inhibitor, broke up super-enhancers by inhibiting *BRD4* and deregulating *MYB* transcription, consequently inhibiting adenoid cystic carcinoma growth (Drier et al., 2016).

This study predicted potential pathogenic super-enhancer-driven genes in PF and built a TF-super-enhancer-associated genes regulatory network. Also, LTBP2 and its super-enhancer binding TF myc were detected to confirm our prediction. Although whether the upregulated DEGs are driven by super-enhancers needs to be confirmed by further experiments, it provides one potential direction to study PF pathogenesis and possible therapeutic targets. Comprehensive analysis of patients’ super-enhancer spectrum or that of healthy people may become an important way of studying the pathological mechanism and disease treatment.

## Data Availability Statement

The datasets presented in this study can be found in online repositories. The names of the repository/repositories and accession number(s) can be found in the article/supplementary material.

## Ethics Statement

The animal study was reviewed and approved by Guangzhou University of Chinese Medicine.

## Author Contributions

HL, YJ, and MZ designed the study. HL and CZ wrote the main manuscript text. HL, CZ, ZL, KY, JZ, and WS performed the experiments. YJ and MZ reviewed the data and conclusions. HL, CZ, MZ, and YJ analyzed the data and prepared the figures. All authors contributed to the article and approved the submitted version.

### Conflict of Interest

The authors declare that the research was conducted in the absence of any commercial or financial relationships that could be construed as a potential conflict of interest.

## Footnotes

^1^https://www.ncbi.nlm.nih.gov/gds

^2^https://version-11-0.string-db.org/

^3^https://cytoscape.org

^4^https://maayanlab.cloud/Enrichr/

^5^https://hiplot.com.cn/
